# The Neutrophil-Lymphocyte Count Ratio in Patients with Community-Acquired Pneumonia

**DOI:** 10.1371/journal.pone.0046561

**Published:** 2012-10-01

**Authors:** Cornelis P. C. de Jager, Peter C. Wever, Eugenie F. A. Gemen, Ron Kusters, Arianne B. van Gageldonk-Lafeber, Tom van der Poll, Robert J. F. Laheij

**Affiliations:** 1 Department of Emergency Medicine and Intensive Care, Jeroen Bosch Ziekenhuis, ’s-Hertogenbosch, The Netherlands; 2 Department of Medical Microbiology and Infection Control, Jeroen Bosch Ziekenhuis, ’s-Hertogenbosch, The Netherlands; 3 Department of Clinical Chemistry and Hematology, Jeroen Bosch Ziekenhuis, ’s-Hertogenbosch, The Netherlands; 4 National Institute for Public Health and the Environment, Bilthoven, The Netherlands; 5 Center of Infection and Immunity Amsterdam and Center of Experimental and Molecular Medicine, Academic Medical Center, University of Amsterdam, The Netherlands; 6 Department of Gastroenterology and Hepatology, University Medical Center Utrecht, Utrecht, The Netherlands; Cardiff University School of Medicine, United Kingdom

## Abstract

**Study Objective:**

The neutrophil-lymphocyte count ratio (NLCR) has been identified as a predictor of bacteremia in medical emergencies. The aim of this study was to investigate the value of the NLCR in patients with community-acquired pneumonia (CAP).

**Methods and Results:**

Consecutive adult patients were prospectively studied. Pneumonia severity (CURB-65 score), clinical characteristics, complications and outcomes were related to the NLCR and compared with C-reactive protein (CRP), neutrophil count, white blood cell (WBC) count. The study cohort consisted of 395 patients diagnosed with CAP. The mean age of the patients was 63.4±16.0 years. 87.6% (346/395) of the patients required hospital admission, 7.8% (31/395) patients were admitted to the Intensive Care Unit (ICU) and 5.8% (23/395) patients of the study cohort died. The NLCR was increased in all patients, predicted adverse medical outcome and consistently increased as the CURB-65 score advanced. NLCR levels (mean ± SD) were significantly higher in non-survivors (23.3±16.8) than in survivors (13.0±11.4). The receiver-operating characteristic (ROC) curve for NLCR predicting mortality showed an area under the curve (AUC) of 0.701. This was better than the AUC for the neutrophil count, WBC count, lymphocyte count and CRP level (0.681, 0.672, 0.630 and 0.565, respectively).

**Conclusion:**

Admission NLCR at the emergency department predicts severity and outcome of CAP with a higher prognostic accuracy as compared with traditional infection markers.

## Introduction

Community-acquired pneumonia (CAP) is a common, potentially fatal disease despite advances in both diagnosis and treatment [1], [2], [3], [4]. Although new techniques are being developed, defining the microbial etiology and classifying the severity of CAP both remain challenging issues. Biomarkers, preferably in combination with clinical risk scores, are increasingly used to identify specific patients at risk, to judge the severity of illness and prognosis of CAP and more recently to guide antibiotic therapy [5], [6], [7], [8], [9]. As the allocation of resources is, however, important, the high prices for the use of newly developed biomarkers make their use less attractive [10].

Immuno-competent white blood cell populations play an important role in the systemic inflammatory response to infection. Following endotoxemia the number of circulating neutrophils increases while lymphocyte counts decrease [11]. Neutrophilia is well recognized as infection marker whereas the clinician is less familiar with absolute lymphocytopenia (lymphocyte count below 1.0×10e9/l) as a possible marker in infectious disease management. Recently, the latter showed its potential in predicting bacteremia or the severity of several infectious diseases [12], [13], [14], [15], [16]. Combining both parameters seems a logical step and the ratio of neutrophil and lymphocyte counts is increasingly used in several clinical circumstances. Initially, this so-called neutrophil-lymphocyte count ratio (NLCR) was studied as an infection marker in ICU patients and found to correlate well with disease severity and outcome, according to APACHE-II and SOFA scores [13], [17], [18]. Other studies focused on the use of the NLCR in specific clinical conditions, like appendicitis, or its use as an independent predictor of survival in patients with various conditions ranging from oncological to cardiovascular diseases [19], [20], [21], [22], [23], [24], [25], [26], [27]. In a retrospective study, the NLCR proved to be a simple and even better marker in predicting bacteremia than routine parameters, like white blood cell (WBC) count and C-reactive protein (CRP) level, in infectious emergency admissions [16]. As CAP is an important reason for Emergency Department (ED) admission and subsequent hospitalization, we prospectively studied the prognostic value of NLCR in patients with this condition.

## Materials and Methods

### Study Design and Setting

From December 2007 to January 2010, consecutive adult (18 years or older) patients admitted to the ED of the Jeroen Bosch Ziekenhuis with suspected CAP were prospectively studied. The Jeroen Bosch Ziekenhuis is an 800-bed teaching hospital in ’s-Hertogenbosch, the Netherlands, with an annual ED census of approximately 28000 visits per year. Clinically suspected CAP was defined as the presence of symptoms of lower respiratory tract infection (new cough, sputum production, dyspnoea, hypo- or hyperthermia, altered breath sounds upon physical examination) in the presence of a new infiltrate on plain chest radiography. Chest radiographs were screened by the ED physician and reviewed by a senior radiologist, unaware of clinical and laboratory findings. Criteria for exclusion were, besides age below 18 years, transferral from another hospital and residence in a nursing home.

### Ethics Statement

The institutional review board approved the study and written informed consent was obtained from the patients or their relatives (local ethics committee, METOPP, Tilburg (NL), number NL 18156.028.07).

### Data Collection and Methods of Measurement

Patient’s characteristics, clinical features and laboratory data were collected and entered in an electronic database. The patients were assessed using available data directly upon admission. There were no patients with HIV/AIDS included in our study. The following data were collected: age, gender, current smoking status, antimicrobial therapy prior to the presentation to the ED, co-morbidity (diabetes mellitus, chronic obstructive pulmonary disease (COPD), heart disease, cancer, gastrointestinal disease, cerebrovascular disease, renal disease and chronic liver disease), additional therapy prior to presentation (pulmonary inhaler therapy, oral corticosteroids), clinical symptoms (mental status, body temperature, blood pressure, heart and respiratory rate, oxygen saturation), laboratory data (CRP level, WBC count, neutrophil count, lymphocyte count, NLCR and urea nitrogen levels) and radiological findings (infiltrate and/or pleural effusion). Biomarkers were measured in all patients as part of routine clinical care.

CRP levels were measured with a fully automated enzyme-linked immuno-assay using an Aeroset 2.0 analyzer (Abbott Diagnostics, Santa Clara, California, USA). The WBC count, neutrophil and lymphocyte counts were determined on a Sysmex XE-2100 hematology analyzer (Sysmex Corporation, Kobe, Japan).

In our hospital, the upper limit of the normal range of the neutrophil count is set at 7.5×10e9/l with a lower limit of the normal range of the lymphocyte count set at 1.0×10e9/l. As previously used for predicting bacteremia, we used the cut-off point of 10.0 for the NLCR to calculate sensitivity, specificity, positive- and negative predicting values [13], [16].

### Microbiological Evaluation

Microbiological evaluation for patients suspected of CAP was performed by sputum culture, aerobic and anaerobic blood cultures, urine immuno-chromatographic antigen detection tests, serum enzyme-linked immunosorbent assays (ELISA) for antibody determination and standard polymerase chain reaction (PCR) techniques for specific pathogens. If necessary, antibody determination was repeated four to eight weeks after admission. Sputum, aerobic and anaerobic blood cultures and the identification of potential pathogenic microorganisms were performed according to standard microbiological methods for detection of among others *Streptococcus pneumoniae, Streptococcus spp, Haemophilus influenzae, Moraxella catarrhalis, Staphylococcus aureus, Pseudomonas aeruginosa, Enterobacteriaceae* and anaerobic bacteria. The presence of *Legionella pneumophila* DNA in sputum and serum was detected by PCR. From 2009 onwards, PCR for *Coxiella burnetii* was performed on serum. With ELISA, sera were tested for the presence of specific IgM and IgG antibodies against *L. pneumophila* serogroups 1–6, *Mycoplasma pneumoniae, Chlamydophila psittaci* and *C. burnetii*. Urine was tested with immunochromatographic antigen detection tests for *L. pneumophila* serogroup 1 and *S. pneumoniae* antigens. A combined nose and throat swab was tested by PCR for influenza A virus, including influenza A (H1N1) from June 2009 onwards, and influenza B virus. Potential pathogenic microorganisms were considered etiologic for CAP when detected in sputum or blood cultures, by PCR or urinary antigen test or in case of seroconversion of specific antibodies.

### Severity of Illness and Outcome

To study the severity of CAP upon presentation, the validated CURB-65 score was calculated in all patients upon admission. The purpose of the CURB-65 score is to calculate the probability of mortality in patients with CAP [28]. The score is based upon five factors from which its name is derived. Risk factors associated with an increased mortality according to the CURB-65 score are: confusion or decreased level of consciousness, abnormal renal function (blood urea nitrogen >7 mmol/L), respiratory frequency ≥30/min, systolic or diastolic blood pressure <90 mmHg or ≤60 mmHg, respectively and age ≥65 years. By this severity score patients were stratified in six risk categories to predict mortality. Furthermore, we assessed hospital or intensive care admissions, length of hospitalization and in-hospital mortality.

### Statistical Analysis

Statistical analysis was performed using SAS, version 9.2 software (SAS Institute Inc, Cary, North Carolina, USA). The level of significance for all statistical tests was a 2-sided, p-value of 0.05. Descriptive analysis was performed for all variables. Student’s t-tests were used to evaluate the differences in CRP levels, WBC counts, neutrophil count, lymphocyte counts and NLCR, adjusted for the distribution of the outcome. To identify differences between the aforementioned markers we performed an unadjusted and adjusted regression analysis and a logistic regression analysis. Receiver operating characteristic (ROC) curves were constructed to evaluate the sensitivity and specificity of CRP level, WBC counts, neutrophil counts and lymphocyte counts and NLCR in predicting survival. ROC curves displayed sensitivity versus 1-specificity such that area under the curves (AUC) varied form 0.5–1.0, with higher values indicating increased discriminatory ability.

## Results

### Patients

During the study period, 562 consecutive patients with the clinical suspicion of CAP were presented at the ED. Because of an alternative diagnosis, 99 patients were excluded.

In 395 (85.3%) out of the initially included 463 patients a new infiltrate was visible on chest radiography and were diagnosed with CAP. A total of 265 patients (67.1%) had an identifiable etiology of CAP.

The mean age of the 395 patients was 63±16 years, with the majority (300, 76%) of the patients being 50 years of age or older. Smoking as a risk factor was noticed in 151 (38%) patients. Co-morbidity was seen in 239 (61%) of the patients, with COPD (131, 33%) reported as the most prominent pre-existing condition (table 1).

**Table 1:** Baseline characteristics upon presentation at the Emergency Department (n = 395).

		Overall	Hospital admission	ICU admission	In-hospital mortality
		(n = 395)	(n = 346)	(n = 31)	(n = 23)
Age	18-49 years	95 (24)	73 (21)	5 (16)	0
	50-64 years	92 (23)	81 (23)	10 (32)	0
	65-74 years	98 (25)	87 (25)	9 (29)	4 (17)
	≥75 years	110 (28)	105 (31)	7 (23)	19 (83)
Gender	Male	240 (61)	210 (61)	20 (65)	15 (65)
Smoking	Yes	151 (38)	129 (37)	15 (48)	5 (22)
Comorbidity	Diabetes mellitus	68 (17)	65 (19)	9 (29)	4 (17)
	Malignancy	49 (12)	45 (13)	4 (13)	2 (9)
	Cardiac	94 (24)	88 (25)	11 (35)	14 (61)
	Cerebrovascular	48 (12)	46 (13)	4 (13)	4 (17)
	Renal	37 (9)	36 (10)	3 (10)	5 (22)
	Hepatic	17 (4)	16 (5)	2 (6)	1 (4)
	COPD	131 (33)	124 (36)	15 (48)	12 (52)
Medication	Oral corticosteroids	53 (13)	48 (14)	4 (13)	6 (26)
	Antibiotics	148 (37)	122 (35)	6 (19)	9 (39)
	Bronchodilators	130 (33)	123 (36)	16 (52)	13 (56)
Pathogen	*S. pneumonia*	73 (18)	66 (19)	14 (45)	5 (22)
	*C. burnetii*	63 (16)	48 (14)	1 (3)	0
	*M. pneumonia*	52 (13)	44 (13)	3 (10)	2 (9)
	*H. influenza*	23 (6)	22 (6)	1 (3)	1 (4)
	Influenza A (H1N1) virus	21 (5)	18 (5)	1 (3)	0

COPD, chronic pulmonary obstructive disease; Chronic Obstructive Pulmonary Disease (COPD) was classified using the global initiative for chronic obstructive lung disease classification (GOLD), Hepatic means liver disease related to malignancy, hepatitis, auto-immune liver disease and/or alcoholic liver disease, cardiac means heart disease related to acute coronary syndrome (cardiovascular disease), valvular disease and/or heart failure, renal means renal disease including current renal replacement therapy, cerebrovascular means cerebrovascular disease; n, number; ICU, intensive care unit; data are presented as number (percentage) of patients.

### Severity of CAP and Infection Markers

The CURB-65 score (mean ± SD) of all patients was 1.6±1.3: 96 patients (24%) had a CURB-65 score 0; 86 patients (22%) a score 1, 115 patients (29%) a score 2; 68 patients (17%) a score 3, 25 patients (6%) a score 4, and 5 patients (1%) a score 5. The CURB-65 score predicted mortality rate of all patients was 5.4% closely resembling the in-hospital mortality in our study (n = 23, 5.8%). Because of the relatively small number of patients with CURB-65 scores 4 and 5, we combined both groups in the data analysis.

**Figure 1:**
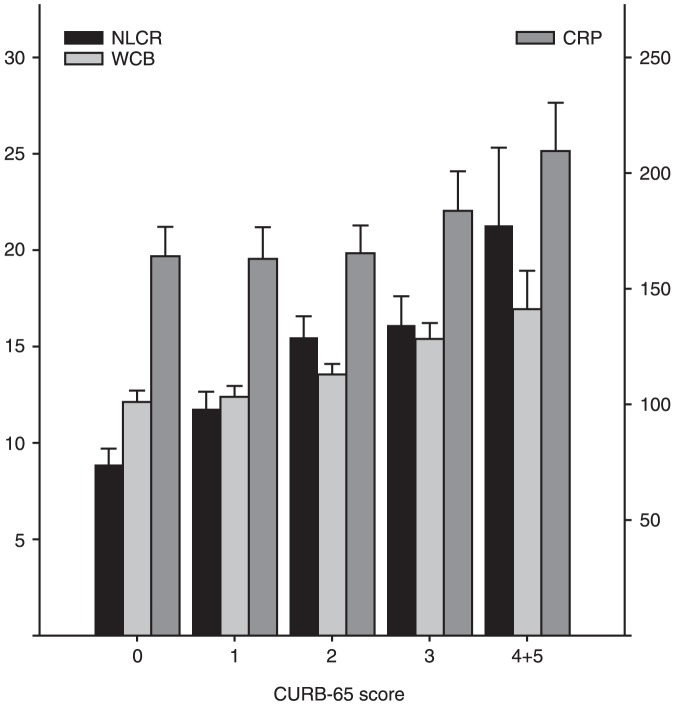
Admission levels of the neutrophil-lymphocyte count ratio, white blood cell count (×10e9/l) and C-reactive protein level (mg/l) in community-acquired pneumonia patients classified into five CURB-65 categories (0, 1, 2, 3 and 4+5), (NLCR, neutrophil-lymphocyte count ratio; WBC, white blood cell; Y-axis left CRP, C-reactive protein; Y-axis right) data are presented as mean and SD.

As the CURB-65 score increased from score 0 to score 4–5, the NLCR consistently increased, while the lymphocyte count consistently decreased. Among patients in different CURB-65 categories there were no significant differences in CRP levels (p = 0.08). Infection markers among patients in different CURB-65 categories are shown in figure 1. Overall, the NLCR (mean ± SD) was increased in patients with CAP (13.6±12.0) and increased even more when patients were admitted to the hospital (14.4±12.4) or ICU (18.7±19.9) or died in-hospital (23.3±16.8). Infection markers of the patients presenting with CAP to the ED are shown in table 2.

**Table 2:** Infection markers in patients admitted to the Emergency Department with community-acquired pneumonia and subpopulations of patients admitted to the hospital and patients deceased during hospitalization.

	CRP (mg/l)	WBC count(10e9/l)	Neutrophil count(10e9/l)	Lymphocyte count(10e9/l)	NLCR
Overall (n = 395)	171 (128)	13.5 (6.5)	11.4 (6.4)	1.1 (0.7)	13.6 (12.0)
Hospital admission (n = 346)	181 (130)	13.9 (6.7)	11.9 (6.5)	1.1 (0.6)	14.4 (12.4)
ICU admission (n = 31)	234 (132)	16.5 (10.8)	14.6 (11.1)	1.1 (0.7)	18.7 (19.9)
In-hospital mortality (n = 23)	142 (118)	16.6 (6.0)	14.4 (5.8)	1.0 (0.8)	23.3 (16.8)

WBC, white blood cell; CRP, C-reactive protein; ICU, intensive care unit; NLCR, neutrophil-lymphocyte count ratio; data are presented as mean (SD).

### Neutrophil-lymphocyte Count Ratio

Data concerning the NLCR are shown in table 3. In patients with an adverse outcome defined as ICU admission and/or mortality (n = 50, 12.7%), a NLCR ≥10.0 was observed significantly more frequent (37/50 (74.0%) versus 13/50 (26.0%) NLCR **<**10, p-value<0.01). No significant differences in NLCR were observed between gender categories in our patients. One third of the patients were previously diagnosed with COPD (n = 131, 33.2%). A significant proportion of these patients showed a NLCR ≥10.0 (85/131 (64.9%) versus 46/131 (35.1%) NLCR <10, p-value <0.01). In patients who had received antibiotics prior to ED admission (n = 148, 37.5%), significantly more patients with a NLCR **<**10 were noted (98/148 (66.2%) versus 50/148 (33.8%) NLCR ≥10.0, p-value <0.01).

**Table 3:** Frequencies of neutrophil-lymphocyte count ratios <10 and ≥10 in subpopulations of patients admitted to the emergency department with community-acquired pneumonia.

		NLCR <10	NLCR ≥10	p-value
		n = 197	n = 198	
Age	18–50 years (n = 95, 24.1%)	60 (30.5)	35 (17.7)	<0.01
	50–64 years (n = 92, 23.3%)	53 (26.9)	39 (19.7)	0.09
	65–74 years (n = 98, 24.8%)	50 (25.4)	48 (24.2)	0.79
	75 year (n = 110, 27.8%)	34 (17.2)	76 (38.4)	<0.01
Gender	Female (n = 155, 39.2%)	72 (46.5)	83 (53.5)	0.27
	Male (n = 240, 60.8%)	125 (52.1)	115 (47.9)	
Co-morbidity	COPD (n = 131, 33.2%)	46 (35.1)	85 (64.9)	<0.01
	Diabetes (n = 68, 17.4%)	28 (41.2)	40 (58.8)	0.11
Medication	Oral corticosteroids (n = 53, 13.4%)	23 (43.4)	30 (56.6)	0.30
	Previous antibiotics (n = 148, 37.5%)	98 (66.2)	50 (33.8)	<0.01
	Bronchodilators (n = 130, 32.9%)	45 (34.6)	85 (65.4)	<0.01
Bacteremia	(n = 42, 10.6%)	9 (21.4)	33 (78.6)	<0.01
Pathogen	*S. pneumoniae* (n = 73, 18.5%)	8 (10.9)	65 (89.1)	<0.01
	*C. burnetii* (n = 63, 15.9%)	49 (77.8)	14 (22.2)	<0.01
	*M. pneumoniae* (n = 52, 13.2%)	32 (61.5)	20 (38.5)	0.07
	*H. influenzae* (n = 23, 5.8%)	11 (47.8)	12 (52.2)	0.83
	Influenza A (H1N1) virus (n = 21, 5.3%)	13 (61.9)	8 (38.1)	0.25
Outcome				
	ICU admission and/or mortality (n = 50, 12.6%)	13 (26.0)	37 (74.0)	<0.01
	ICU admission (n = 31, 7.8%)	9 (29.0)	22 (71.0)	<0.01
	In-hospital mortality (n = 23, 5.8.%)	5 (21.7)	18 (78.3)	<0.01

NLCR, neutrophil-lymphocyte count ratio; COPD, chronic pulmonary obstructive disease; ICU, intensive care unit; n, number; data are presented as number (percentage) of patients.

### Microbiology

Sputa, blood culture samples, combined nose and throat swabs, urine samples for antigen testing and sera for serology were obtained in 299, 378, 344, 348 and 384 patients, respectively.

Upon presentation, 148 patients (37.5%) were using antibiotics (prescribed by the general physician). In 265 patients (67.1%) the microbial etiology could be identified. A single pathogen was detected in 208 patients and two or more pathogens in 57 patients. The most frequently identified pathogens were *S. pneumoniae* (n = 73, 18.5%), *C. burnetii* (n = 63, 15.9%), *M. pneumoniae* (n = 52, 13.2%), *H. influenzae* (n = 23, 5.8%) and influenza A H1N1 virus (n = 21, 5.3%). Positive blood cultures were seen in 42 (10.6%) patients. An overview of isolated pathogens is shown in table 4 (all microbiological results).

**Table 4:** Causative microorganisms of community-acquired pneumonia in the study patients (n = 395).

Microorganism	Number (%)
*Streptococcus pneumoniae*	73 (18.5)
*Coxiella burnetii*	63 (15.9)
*Mycoplasma pneumoniae*	52 (13.2)
*Haemophilus influenzae*	23 (5.8)
Influenza A (H1N1) virus	21 (5.3)
*Staphylococcus aureus*	12 (3.0)
*Legionella pneumophila*	7 (1.7)
*Mycobacterium tuberculosis*	3 (0.7)
*Chlamydia psittaci*	2 (0.5)
*Klebsiella pneumoniae*	2 (0.5)
*Moraxella catarrhalis*	7 (1.7)
Other gram-negative	7 (1.7)
None	130 (32.9)

Patients with *S. pneumoniae* infection significantly more often had a NLCR ≥10.0 (65/73 (89.1%) versus 8/73 (10.9%) NLCR **<**10, p-value <0.01). In contrast, patients with *C. burnetii*-related CAP significantly more often showed a NLCR value **<**10 (49/63 (77.8%) versus 14/63 (22.2%) NLCR ≥10.0, p-value <0.01). The mean CURB-65 score in patients with CAP due to *S. pneumoniae* was higher than in patients with CAP due to *C. burnetii* (2.0±1.2 versus 1.0±1.2, p-value <0.01). Patients with *M. pneumoniae*, *H. influenzae* or influenza H1N1 virus-related CAP also more often showed a NLCR **<**10 (table 3). Adjusted for the CURB-65 score the proportion of patients with a NLCR ≥10.0 was higher in patients with *S. pneumoniae* infection (p-value <0.01) whereas most patients with a CAP due to *C. burnetii* had a NLCR **<**10 (p-value <0.01).

Patients with clinically significant positive blood cultures (n = 42, 10.6%) had significantly higher NLCR values compared to patients with negative blood cultures (22.5±19.0 versus 12.6±10.4, p-value <0.01). WBC count (15.9±5.5 versus 13.2±6.6×10e9/l, p-value <0.01) and neutrophil count (14.4±6.0 versus 11.1±6.3×10e9/l, p<0.01) were also both significantly higher in bacteremic patients. The lymphocyte count was significantly lower in patients with positive blood cultures compared to patients with negative blood cultures (0.9±0.5 versus 1.2±0.7×10e9/l, p-value <0.01). In contrast, CRP showed no statistical significant difference between bacteremic and non-bacteremic patients (205±141 versus 166±127 mg/l, p-value = 0.07). Significantly more patients with positive blood cultures had a NLCR ≥10.0 (33/42 (78.6%) versus 9/42 (21.4%) NLCR **<**10, p-value <0.01).

### Hospitalization and Mortality

Overall, 346 patients (88%) were hospitalized with a mean stay of 10.9±11.7 days. In all 31 (7.8%) patients were admitted to the ICU. Patients hospitalized for more than 10 days (n = 104, 26.3%) showed significantly higher NLCR values at presentation compared to patients who were not hospitalized or hospitalized for less than 10 days (16.9±15.4 versus 12.4±10.3, p-value <0.01). A total of 23 (5.8%) patients did not survive hospitalization. In patients who died there was a significantly higher NLCR at presentation compared to patients that survived (23.3±16.8 versus 13.0±11.4, p-value <0.01). Both WBC count (16.6±6.0 versus 13.3±6.5×10e9/l, p = 0.02) and neutrophil count (14.4±5.8 versus 11.2±6.4×10e9/l, p = 0.02) showed significant differences between non-survivors and survivors. There was no statistically significant difference in non-survivors versus survivors with respect to the lymphocyte count (1.0±0.8 versus 1.2±0.7×10e9/l) and CRP level (142±119 versus 173±129 mg/l; table 2).

**Figure 2:**
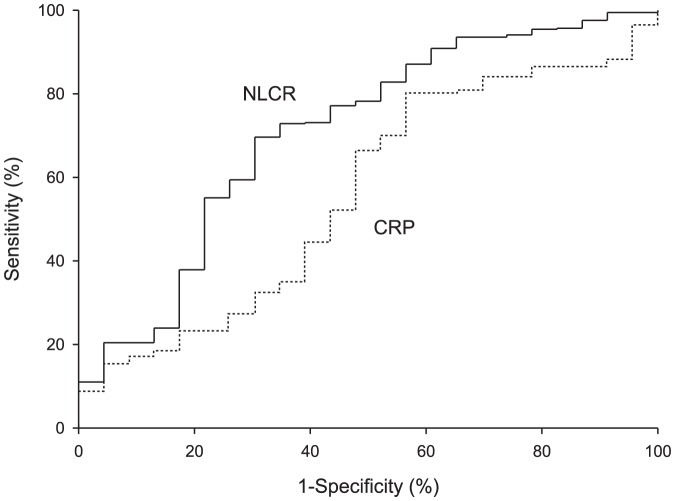
Receiver operating characteristic curves comparing C-reactive protein and the neutrophil-lymphocyte count ratio with respect to prediction of death (NLCR, neutrophil-lymphocyte count ratio; CRP, C-reactive protein).

The NLCR predicted mortality better as compared to CRP level, WBC count, neutrophil count and lymphocyte count. The receiver operating characteristic (ROC) curves of the NLCR and CRP for differentiating survival versus non-survival are presented in figure 2. The NLCR had the highest area under the curve (AUC) of 0.701, followed subsequently by the AUC of the neutrophil count, WBC count, lymphocyte count and CRP level (0.681, 0.672, 0.630 and 0.565, respectively). Although the AUROC of the NLCR (0.701) is more prominent than the AUROC of the CRP level (0.565), the moderate added value considering the AUROC of the neutrophil count (0.680) may well reflect differences in neutrophil numbers. Unadjusted logistic regression analysis on the prediction of mortality shows that the NLCR is a better predictor of mortality than the neutrophil count (p<0.05; the regression coefficients estimates for neutrophils and NLCR were 0.01 (p value 0.85) and 0.04 (p value 0.01) respectively. Adjusted for differences in baseline characteristics between patients who died and those who did not, the regression coefficients estimates for neutrophils and NLCR were 0.03 (p value 0.59) and 0.02 (p value 0.34) respectively.

Multivariate analysis has been performed to determine baseline factors independently associated with poor outcome. Age and heart failure were associated with poor outcome. Nevertheless there was no significant difference between the adjusted and unadjusted logistic regression models examining the prediction of mortality using the NLCR.

## Discussion

Recently, the NLCR has been “rediscovered” as a simple, promising marker in several clinical circumstances. This is the first study that further explored the potential of this infection marker in patients with CAP in an emergency care setting. The discriminatory capacity of the NLCR in CAP patients outweighed predictive values of traditional biomarkers. Increased NLCR values were seen in patients with increased CURB-65 scores, positive blood cultures and unfavourable clinical outcome (prolonged hospitalization, ICU admission and/or death). The AUC of the NLCR ROC curve was significantly higher than that of conventional markers, especially CRP, in predicting mortality in CAP patients.

The host inflammatory response in the development of pneumonia has gained growing interest and infection markers are increasingly used to facilitate treatment decisions and improve the accuracy of clinical severity scores in patients admitted with CAP [3], [5], [6], [7], [29], [30]. “Old” markers like CRP, WBC count and neutrophil count are still the most frequently used infection markers in daily clinical practice [31]. Although recently introduced infection markers such as procalcitonin, several cytokines and markers like endothelin-1, copeptin and pro-adrenomedullin show promising results in risk assessment and outcome prediction the implementation of these “new” infection markers is hampered by validation, costs and accessibility.

In various stressful events the physiological response of circulating leucocytes is characterized by an increase in neutrophil counts and a decline in lymphocyte counts. Neutrophilia is caused by demargination of neutrophils, delayed apoptosis of neutrophils and stimulation of stem cells by growth factors. Margination of lymphocytes, redistribution of lymphocytes and marked accelerated apoptosis are supposed mechanisms of the observed lymphocytopenia in infectious emergencies [13], [32], [33], [34], [35]. Lymphocytopenia has shown promising results in the prediction of bacteremia in infectious emergency admissions [12], [14], [16], [30]. Although relatively unknown as a marker of disease severity or prognosis, lymphocytopenia has been described in several forms of CAP, especially in the acute phase and probably limited to T-cells and T-cell subsets [36]. In CAP patients it is hypothesized that depression of absolute peripheral blood T-cell counts represents the shift of these cells towards the lung in order to be sequestered in protective mechanisms [37], [38]. The mean lymphocyte count in our overall study population was just above the lower limit of normal, and virtually identical to a figure previously reported in a group of 94 patients with pneumonia [36].

Goodman *et al* initially described the ratio of neutrophil and lymphocyte counts in a retrospective study to assess its potential in diagnosing appendicitis [27]. Besides the prognostic capacity of the NLCR in predicting survival in patients with several oncological diseases and as a prognostic parameter in cardiovascular medicine, Zahorec *et al* further explored its use as a marker of systemic inflammation [13], [19], [22], [23], [24], [25], [26], [27], [39]. Recently, we showed that the NLCR proved to be a simple infection marker with discriminatory capacity in predicting bacteremia in infectious emergency admissions as compared to CRP level, neutrophil count and WBC count [16].

In the current prospective study, we further explored the value of the NLCR in patients admitted with CAP. Interestingly, patients with *S. pneumoniae*, which was the most frequently isolated pathogen in our study, had the highest frequency of increased NLCR values compared to patients with other pathogens. This could be well related to the severity of disease in these patients. Patients with pneumococcal pneumonia tend to be sicker as reflected in increased duration of hospitalization, ICU admittance and mortality. Indeed, in our study CURB-65 scores in patients with pneumococcal infection were higher, while nearly one quarter of the non-surviving patients were diagnosed with pneumococcal disease. Our current study adds to the value of the NLCR by showing that this marker is of interest in patients admitted to the ED with CAP. In our opinion the novelty of the NLCR is the possibility of implementing this parameter simply by using already available biomarkers (WBC-count, neutrophil count and lymphocyte count). Since calculating the NLCR is easy to do and does not require additional testing it may add to our ability to predict mortality. Diagnosing community-acquired pneumonia and subsequently assessing prognosis, severity and site-of-care indicators remains a challenging process. Clinical judgement remains the cornerstone to determine appropriate management but may be facilitated (especially for the less experienced doctors) by the use of severity scores and to a lesser extent biomarkers. [40] It could be of interest to investigate whether adding the NLCR to currently existing severity scores would improve the overall performance of these scores thereby assisting the emergency physician in the treatment options. Use of the NLCR may allow the clinician to stratify patients with CAP into different prognostic categories and could possibly add to the performance of well-accepted severity-of-illness scores.

This study has several limitations. First, in view of the minor differences between the AUC for neutrophil numbers and NLCR, the NLCR may simply reflect differences in neutrophil numbers. Second, as this is a single centre study the results should be validated in other settings. Third, recently developed infection markers (procalcitonin, pro-adrenomedullin, neopterin) were not evaluated. Fourth, in general biomarkers alone are clearly less suited in the prediction of prognosis and severity of disease. Several severity scores are currently used, all with different strengths and weaknesses. In our hospital the CURB-65 is employed because of its ease of use. Considering the mean age of our patients (63 years) the Pneumonia Severity Index (PSI) was probably better suited and it would have been of interest to see the relation of the NLCR and the different PSI categories. In addition the CURB-65 is known for being poor at predicting ICU admission as opposed to mortality. [41] Fifth, the epidemiology of CAP is subject to epidemic outbreaks. During the study period, we were confronted with epidemics of two different pathogens, a regional Q fever epidemic and the influenza A (H1N1) 2009 pandemic. C. burnetii was the second most common cause of CAP in our study but has not been a significant pathogen in most other etiologic studies. The clinical symptoms of CAP due to C. burnetii tend to be less severe compared to other forms of CAP. Previously, we have shown that acute Q fever specifically induces an increase in CRP levels while WBC counts remain within normal ranges [42]. Consequently, this may have influenced the results of our study. Sixth, previously, the NLCR has shown its value both in predicting bacteremia and survival in various clinical circumstances. In general, blood cultures in CAP patients have a relatively low positivity rate. This was also seen in our study (n = 42 patients, 10.6%) and possibly related to the prescription of antibiotics before presentation to the ED (n = 148, 37.5%). Consequently, the low number of actual bacteraemia patients may have affected the final outcomes of our study.

The proportion of patients that required ICU admission that were given antibiotics is clearly decreased compared to the proportion of the non-ICU patients receiving antibiotics. Delayed antibiotic therapy in septic patients is known for its detrimental effects however the antibiotics usage referred to in our study refers to this treatment prior to the ED admission. [43], [44] Detailed examination of the patients admitted to the intensive care shows that although there is a decreased proportion of patients receiving antibiotics prior to admission compared to the overall population, this is not related to survival. A closer look into the patients that actually died showed that the actual number of patients that received antibiotic prior to admission is comparable to the overall study population (39% versus 37%) and that there was no significant differences in survival between the group of patients receiving antibiotics versus the group of patients that did not receive antibiotics prior to the admission (logistic regression analysis, p = 0,86).

## References

[1] Mandell LA, Wunderink RG, Anzueto A, Bartlett JG, Campbell GD, et al (2007) Infectious Diseases Society of America/American Thoracic Society consensus guidelines on the management of community-acquired pneumonia in adults. Clin Infect Dis 44 Suppl 2 S27–72. 17278083 10.1086/511159 PMC7107997

[2] Garau J, Baquero F, Perez-Trallero E, Perez JL, Martin-Sanchez AM, et al (2008) Factors impacting on length of stay and mortality of community-acquired pneumonia. Clin Microbiol Infect 14: 322–329. 18190569 10.1111/j.1469-0691.2007.01915.x

[3] Polverino E, Torres Marti A (2011) Community-acquired pneumonia. Minerva Anestesiol 77: 196–211. 21242952

[4] Fine MJ, Stone RA, Singer DE, Coley CM, Marrie TJ, et al (1999) Processes and outcomes of care for patients with community-acquired pneumonia: results from the Pneumonia Patient Outcomes Research Team (PORT) cohort study. Arch Intern Med 159: 970–980. 10326939 10.1001/archinte.159.9.970

[5] Christ-Crain M, Muller B (2007) Biomarkers in respiratory tract infections: diagnostic guides to antibiotic prescription, prognostic markers and mediators. Eur Respir J 30: 556–573. 17766633 10.1183/09031936.00166106

[6] Muller B, Harbarth S, Stolz D, Bingisser R, Mueller C, et al (2007) Diagnostic and prognostic accuracy of clinical and laboratory parameters in community-acquired pneumonia. BMC Infect Dis 7: 10. 17335562 10.1186/1471-2334-7-10 PMC1821031

[7] Schuetz P, Wolbers M, Christ-Crain M, Thomann R, Falconnier C, et al (2010) Prohormones for prediction of adverse medical outcome in community-acquired pneumonia and lower respiratory tract infections. Crit Care 14: R106. 20529344 10.1186/cc9055 PMC2911752

[8] Kopterides P, Siempos, II, Tsangaris I, Tsantes A, Armaganidis A (2010) Procalcitonin-guided algorithms of antibiotic therapy in the intensive care unit: a systematic review and meta-analysis of randomized controlled trials. Crit Care Med 38: 2229–2241. 20729729 10.1097/CCM.0b013e3181f17bf9

[9] Schuetz P, Christ-Crain M, Muller B (2007) Biomarkers to improve diagnostic and prognostic accuracy in systemic infections. Curr Opin Crit Care 13: 578–585. 17762239 10.1097/MCC.0b013e3282c9ac2a

[10] Heyland DK, Johnson AP, Reynolds SC, Muscedere J (2011) Procalcitonin for reduced antibiotic exposure in the critical care setting: A systematic review and an economic evaluation. Crit Care Med 39: 1792–1799. 21358400 10.1097/CCM.0b013e31821201a5

[11] Jilma B, Blann A, Pernerstorfer T, Stohlawetz P, Eichler HG, et al (1999) Regulation of adhesion molecules during human endotoxemia. No acute effects of aspirin. Am J Respir Crit Care Med 159: 857–863. 10051263 10.1164/ajrccm.159.3.9805087

[12] Wyllie DH, Bowler IC, Peto TE (2005) Bacteraemia prediction in emergency medical admissions: role of C reactive protein. J Clin Pathol 58: 352–356. 15790696 10.1136/jcp.2004.022293 PMC1770625

[13] Zahorec (2001) Ratio of neutrophil to lymphocyte counts-rapid and simple parameter of systemic inflammatiion and stress in critically ill. Bratisl Lek Listy 102: 5–14. 11723675

[14] Wyllie DH, Bowler IC, Peto TE (2004) Relation between lymphopenia and bacteraemia in UK adults with medical emergencies. J Clin Pathol 57: 950–955. 15333656 10.1136/jcp.2004.017335 PMC1770434

[15] Hawkins CA, Collignon P, Adams DN, Bowden FJ, Cook MC (2006) Profound lymphopenia and bacteraemia. Intern Med J 36: 385–388. 16732866 10.1111/j.1445-5994.2006.01076.x

[16] de Jager CP, van Wijk PT, Mathoera RB, de Jongh-Leuvenink J, van der Poll T, et al (2010) Lymphocytopenia and neutrophil-lymphocyte count ratio predict bacteremia better than conventional infection markers in an emergency care unit. Crit Care 14: R192. 21034463 10.1186/cc9309 PMC3219299

[17] Knaus WA, Draper EA, Wagner DP, Zimmerman JE (1985) APACHE II: a severity of disease classification system. Crit Care Med 13: 818–829. 3928249

[18] Vincent JL, Moreno R, Takala J, Willatts S, De Mendonca A, et al (1996) The SOFA (Sepsis-related Organ Failure Assessment) score to describe organ dysfunction/failure. On behalf of the Working Group on Sepsis-Related Problems of the European Society of Intensive Care Medicine. Intensive Care Med 22: 707–710. 8844239 10.1007/BF01709751

[19] Ommen SR, Hodge DO, Rodeheffer RJ, McGregor CG, Thomson SP, et al (1998) Predictive power of the relative lymphocyte concentration in patients with advanced heart failure. Circulation 97: 19–22. 9443426 10.1161/01.cir.97.1.19

[20] Acanfora D, Gheorghiade M, Trojano L, Furgi G, Pasini E, et al (2001) Relative lymphocyte count: a prognostic indicator of mortality in elderly patients with congestive heart failure. Am Heart J 142: 167–173. 11431674 10.1067/mhj.2001.115792

[21] Huehnergarth KV, Mozaffarian D, Sullivan MD, Crane BA, Wilkinson CW, et al (2005) Usefulness of relative lymphocyte count as an independent predictor of death/urgent transplant in heart failure. Am J Cardiol 95: 1492–1495. 15950581 10.1016/j.amjcard.2005.02.022

[22] Gibson PH, Croal BL, Cuthbertson BH, Small GR, Ifezulike AI, et al (2007) Preoperative neutrophil-lymphocyte ratio and outcome from coronary artery bypass grafting. Am Heart J 154: 995–1002. 17967611 10.1016/j.ahj.2007.06.043

[23] Halazun KJ, Aldoori A, Malik HZ, Al-Mukhtar A, Prasad KR, et al (2008) Elevated preoperative neutrophil to lymphocyte ratio predicts survival following hepatic resection for colorectal liver metastases. Eur J Surg Oncol 34: 55–60. 17448623 10.1016/j.ejso.2007.02.014

[24] Tamhane UU, Aneja S, Montgomery D, Rogers EK, Eagle KA, et al (2008) Association between admission neutrophil to lymphocyte ratio and outcomes in patients with acute coronary syndrome. Am J Cardiol 102: 653–657. 18773982 10.1016/j.amjcard.2008.05.006

[25] Halazun KJ, Hardy MA, Rana AA, Woodland DCt, Luyten EJ, et al (2009) Negative impact of neutrophil-lymphocyte ratio on outcome after liver transplantation for hepatocellular carcinoma. Ann Surg 250: 141–151. 19561458 10.1097/SLA.0b013e3181a77e59

[26] Sarraf KM, Belcher E, Raevsky E, Nicholson AG, Goldstraw P, et al (2009) Neutrophil/lymphocyte ratio and its association with survival after complete resection in non-small cell lung cancer. J Thorac Cardiovasc Surg 137: 425–428. 19185164 10.1016/j.jtcvs.2008.05.046

[27] Goodman DA, Goodman CB, Monk JS (1995) Use of the neutrophil:lymphocyte ratio in the diagnosis of appendicitis. Am Surg 61: 257–259. 7887542

[28] Lim WS, van der Eerden MM, Laing R, Boersma WG, Karalus N, et al (2003) Defining community acquired pneumonia severity on presentation to hospital: an international derivation and validation study. Thorax 58: 377–382. 12728155 10.1136/thorax.58.5.377 PMC1746657

[29] Martinez R, Menendez R, Reyes S, Polverino E, Cilloniz C, et al (2011) Factors associated with inflammatory cytokine patterns in community-acquired pneumonia. Eur Respir J 37: 393–399. 20595152 10.1183/09031936.00040710

[30] Chalupa P, Beran O, Herwald H, Kasprikova N, Holub M (2011) Evaluation of potential biomarkers for the discrimination of bacterial and viral infections. Infection. 10.1007/s15010-011-0126-4 21720792

[31] Adams NG (2005) Diagnostic use of C-reactive protein in bacteraemic emergency department patients. Emerg Med Australas 17: 371–375. 16091100 10.1111/j.1742-6723.2005.00759.x

[32] Joshi VD, Kalvakolanu DV, Cross AS (2003) Simultaneous activation of apoptosis and inflammation in pathogenesis of septic shock: a hypothesis. FEBS Lett 555: 180–184. 14644412 10.1016/s0014-5793(03)01271-7

[33] Ayala A, Herdon CD, Lehman DL, Ayala CA, Chaudry IH (1996) Differential induction of apoptosis in lymphoid tissues during sepsis: variation in onset, frequency, and the nature of the mediators. Blood 87: 4261–4275. 8639785

[34] Unsinger J, Kazama H, McDonough JS, Hotchkiss RS, Ferguson TA (2009) Differential lymphopenia-induced homeostatic proliferation for CD4+ and CD8+ T cells following septic injury. J Leukoc Biol 85: 382–390. 19088177 10.1189/jlb.0808491 PMC2653946

[35] Le Tulzo Y, Pangault C, Gacouin A, Guilloux V, Tribut O, et al (2002) Early circulating lymphocyte apoptosis in human septic shock is associated with poor outcome. Shock 18: 487–494. 12462554 10.1097/00024382-200212000-00001

[36] Fantin B, Joly V, Elbim C, Golmard JL, Gougerot-Pocidalo MA, et al (1996) Lymphocyte subset counts during the course of community-acquired pneumonia: evolution according to age, human immunodeficiency virus status, and etiologic microorganisms. Clin Infect Dis 22: 1096–1098. 8783719 10.1093/clinids/22.6.1096

[37] Williams RC Jr, Koster FT, Kilpatrick KA (1983) Alterations in lymphocyte cell surface markers during various human infections. Am J Med 75: 807–816. 6605684 10.1016/0002-9343(83)90412-6

[38] Laurence J (1993) T-cell subsets in health, infectious disease, and idiopathic CD4+ T lymphocytopenia. Ann Intern Med 119: 55–62. 8098929 10.7326/0003-4819-119-1-199307010-00010

[39] Walsh SR, Cook EJ, Goulder F, Justin TA, Keeling NJ (2005) Neutrophil-lymphocyte ratio as a prognostic factor in colorectal cancer. J Surg Oncol 91: 181–184. 16118772 10.1002/jso.20329

[40] Blasi F, Bocchino M, Di Marco F, Richeldi L, Aliberti S (2012) The role of biomarkers in low respiratory tract infections. Eur J Intern Med 23: 429–435. 22726371 10.1016/j.ejim.2012.05.002

[41] Buising KL, Thursky KA, Black JF, MacGregor L, Street AC, et al (2006) A prospective comparison of severity scores for identifying patients with severe community acquired pneumonia: reconsidering what is meant by severe pneumonia. Thorax 61: 419–424. 16449258 10.1136/thx.2005.051326 PMC2111174

[42] de Wit NC, de Jager CP, Meekelenkamp JC, Schoorl M, van Gageldonk-Lafeber AB, et al (2009) Markers of infection in inpatients and outpatients with acute Q-fever. Clin Chem Lab Med 47: 1407–1409. 19778289 10.1515/CCLM.2009.307

[43] Kumar A, Roberts D, Wood KE, Light B, Parrillo JE, et al (2006) Duration of hypotension before initiation of effective antimicrobial therapy is the critical determinant of survival in human septic shock. Crit Care Med 34: 1589–1596. 16625125 10.1097/01.CCM.0000217961.75225.E9

[44] Dellinger RP, Levy MM, Carlet JM, Bion J, Parker MM, et al (2008) Surviving Sepsis Campaign: international guidelines for management of severe sepsis and septic shock: 2008. Crit Care Med 36: 296–327. 18158437 10.1097/01.CCM.0000298158.12101.41

